# 

**Published:** 1903-01-24

**Authors:** 


### NOTICE TO CORRESPONDENTS.

All MSS., Letters, Books for Review, and other matters intended for the Editor should be addressed THE EDITOR, "THE HOSPITAL," THE HOSPITAL BUILDING, 28 & 29 SOUTHAMPTON STREET, STRAND, W.C.

The Editor cannot undertake to return rejected MS., even when accompanied by stamped directed envelope.

### Notice to Advertisers and Subscribers.

All advertisements Orders for Copies of THE HOSPITAL, and Business Communications should be addressed to **THE MANAGER, (Not to the Editor)** THE HOSPITAL, 28 & 29 Southampton Street, Strand London, W.C.

The Cost of Subscription, post free, is as follows.—Per week, 2½d.; per quarter, 2s. 8d.; per half-year, 5s. 3d.; per annum, 10s. 6d. Foreign:—Per annum, 15s. 2d., payable, in all cases, in advance.

**NOTICE.—Vol. XXXII. of "The Hospital" (April to September 1902) in handsome cloth binding is now on sale at the office of this Journal, price, 7s. 6d. Cases for binding the Numbers contained in this Volume 1s. 6d. Index to Vol. XXXII., 2½d., post free.**

**The Monthly Part for January is now ready. Price 6d., by post 8d.**

### BOOKS RECEIVED.

Baillière, Tindall and Cox.

"Points of Practical Interest in Gynæcology." By H. Macnaughton-Jones, M.D.

"The Prize Essay on the Erection of the King Edward VII. Sanatorium for Consumption." By Arthur Latham, M.D., and A. William West.

"Constipation." By Sherman, Bigg, F.R.C.S.E.

His Majesty's Stationery Office.

"Thirty-First Annual Report of the Local Government Board, 1901–1902."

National Anti-vivisection Society.

"The Metropolitan Hospitals and Vivisection."

W. B. Saunders and Co.

"The Four Epochs of a Woman's Life." By Anna M. Galbraith, M.D.

"Personal Hygiene." By Walter L. Pyle, M.D.

Sands and Co.

"The Army Medical System: What it is, is not, and ought to be."

G. T. Buggeley, Newcastle.

"A Concise History of the North Staffordshire Infirmary." By Ralph Hoadley.

University Tutorial Press.

"London University Tutorial Guide, and University Correspondence College Calendar, 1902–3."

J. W. Arrowsmith, Bristol.

"The Reputation of the Hotwells (Bristol) as a Health Resort." By G. L. M. Griffiths.

H. Froude.

"The Hospital Service Book." By Rev. C. Parkhurst Baxter, M.A.

Liebig Extract of Meat Co.

"Influenza." By R. Bell, M.D.

Edward Lloyd.

"The London Manual, 1903." Edited by Robert Donald.

J. Wright and Co.

"Golden Rules of Refraction." By Ernest E. Maddox, M.D.

### SCRAPS AND GLEANINGS.

THE Society for the Propagation of the Gospel in Foreign Parts has received two anonymous donations of £500 each.

THE Princess Christian gave her patronage to an amateur performance of "The Geisha" which was recently given at the Shaftesbury Theatre in aid of the Church of England Waifs and Strays.

DURING the Parliamentary Session just closed, 2,563,564 letters passed through the lobby post-office, of which 1,565,270 were received; 54,354 private telegrams were sent and received, while the Press telegrams numbered 45,704.

SIR JOHN LENG, M.P., will preside at the sixty-fourth anniversary festival dinner of the Newsvendors' Benevolent and Provident Institution, to be held in London, May, 1903. Membership of this old institution is open to all persons engaged in the sale of newspapers throughout the United Kingdom.

### THE STUDENT OF MEDICINE.

### APPOINTMENTS.

**H. C. P. Bennett**, M.B.Lond., appointed House Physician to the Auckland Hospital, New Zealand. **Hugh Howie Borland**, M.D., C.M., D.P.H Camb., appointed Vaccinator to the Glasgow Royal Infirmary. **Edmund R. Branch**, M.B., Ch.B.Edin., appointed District Medical Officer at St. Kitts. **Ada M. Browne**, L.S.A., appointed Anæsthetist to the New Hospital for Women, Euston Road. **Charles M. Deane**, M.D., M.R.C.S., appointed Port Health Officer and Medical Officer to Police, Gaols, and Paupers, Strahan, Tasmania. **Frederick Dittmar**, M.D., C.M. Glasg., D.P H.Camb., appointed Medical Officer of Health for Scarborough. **John James Edgar**, M.B., C.M.Glasg., L.R.C.P. and S.Edin., appointed Public Vac inator for Napier, Tasmania. **G. E. A. Evans**, M.R.C.S., L.R.C.P.Lond., appointed Medical Officer of the Branscombe District of the Honiton Union. **P. J. Garland**, L.R.C.P., L.R.C.S.Irel., appointed Senior Medical Officer of the Gold Coast Colony. **Dudley Greaves**, L.R.C.P., L.R.C.S.Edin., L.F.P.S.Glasg., appointed District Medical Officer at Dominica. **Otto Grunbaum**, M.A., M.B., B C.Cantab., M.R.C.P.Lond., D.Sc.Lond., appointed Medical Registrar at the London Hospital. **Robert Arthur Lyster**, M.B., B.Ch., B.Sc., D.P.H., appointed Assistant to the Chair of Hygiene and Public Health in the University of Birmingham. **Alan Muir**, M.B., C.M.Glasg., appointed Medical Officer to the Kyle Union Poorhouse Board. **J. Horace Palmer**, L.R.C.P., L.R.C.S.E., L.F.P. and S.G., L.M., appointed Medical Officer and Public Vaccinator to the Gamlingay District of the Caxton and Arrington Union. **A. W. Rendor**, M.R.C.P., L.R.C S.Edin., appointed Surgeon to the Broad Arrow Hospital, Western Australia. **G. M. Scott**, M.D., B.C.Camb., appointed Honorary Ophthalmic Surgeon to the Kalgoorlie Hospital, Western Australia. **John Cecil Toswill**, M.B., C.M.Edin., appointed Public Vaccinator at Hastings, Tasmania.

**"The Hospital" Institutional Library.**

"Hospitals and Asylums of the World." 4 Vols., with a Portfolio of Plans, complete £12 12s.
"Burdett's Hospitals and Charities, 1902." 5s.
"Cottage Hospitals, General, Fever, and Convalescent." 10s. 6d.
"Hospital Expenditure: The Commissariat." 2s. 6d.
"A Legal Handbook for the Use of Hospital Authorities." 2s. 6d.
"The Cottage Hospital Case Book or Register of Patients." 10s. and 12s. 6d.
"The Furnishing and Appliances of a Cottage Hospital." 6d.
All these are published by the SCIENTIFIC PRESS, Ltd., and may be obtained through any bookseller, or direct from the publishers, 28 and 29 Southampton Street, Strand, London, W.C.

### Useful Books for Nurses' Libraries.

### THE NURSING PROFESSION: HOW AND WHERE TO TRAIN.

Being a Guide to Training for the Calling of a Nurse.

Edited by Sir HENRY BURDETT, K.C.B.

Crown 8vo., 2/- net, 2/4 post free.

### A HANDBOOK FOR NURSES.

By J. K. WATSON, M.D., M B., C.M.

New and Revised Edition.

Crown 8vo., profusely illustrated, cloth gilt.

5/- net, 5/4 post free.

### NURSING: ITS THEORY AND PRACTICE.

Being a complete Text-book of Medical, Surgical, and Monthly Nursing.

By PERCY C. LEWIS, M.D., M.R.C.S., L.S.A.

New Edition, Enlarged and Revised (17th thousand), profusely illustrated with over 100 new Cuts. Crown 8vo., cloth gilt.

3/6 post free.

### NURSING: ITS PRINCIPLES AND PRACTICE.

By ISABEL ADAMS HAMPTON.

New and Enlarged Edition.

Crown 8vo., illustrated, cloth gilt.

7/6 net, 7/10 post free.

### SURGICAL WARD-WORK AND NURSING.

By ALEXANDER MILES, M.D.Edin., C.M., F.R.C.S.E.

Second Edition, Enlarged and entirely Revised.

Demy 8vo., copiously illustrated, cloth gilt.

3/6 net, 3/10 post free.

### ELEMENTARY ANATOMY AND SURGERY FOR NURSES.

By WILLIAM McADAM ECCLES, M.S.Lond., M.B., F.R.C.S., L.R.C.P.

Crown 8vo., profusely illustrated, Cloth. 2/6 post free.

### ELEMENTARY PHYSIOLOGY FOR NURSES.

By C. F. MARSHALL, M.D., B.Sc., F.R.C.S.

Crown 8vo., illustrated.

Cloth. 2/- post free.

### THE NURSE'S DICTIONARY OF MEDICAL TERMS AND NURSING TREATMENT.

Compiled for the Use of Nurses.

By HONNOR MORTEN.

Fourth and Revised Edition (50th thousand). Demy 16mo. (suitable for the apron pocket).
Bound in cloth boards, 160 pp., 2/- post free.
In handsome leather gilt, 2/6 net, 2/8 post free.

### HOSPITAL SISTERS AND THEIR DUTIES.

By EVA C. S. LÜCKES, Matron, London Hospital.

Third Edition, enlarged and partly re-written.

Crown 8vo., about 200 pp. cloth gilt.

2/6 post free.

### OPHTHALMIC NURSING.

By SYDNEY STEPHENSON, M.B., F.R.C.S.E.

New and Revised Edition.

Crown 8vo., profusely illustrated with Original Drawings, List of Instruments required, and a Glossary of Terms. Cloth.

3/6 net, 3/10 post free.

### ART OF MASSAGE.

By Mrs. CREIGHTON HALE.

Second Edition.

Demy 8vo., 150 pp., profusely illustrated with over 70 Special Cuts.

Cloth gilt,

6/- post free.

### A PRACTICAL HANDBOOK OF MIDWIFERY.

By FRANCIS W. HAULTAIN, M.D.

Now Ready.

New and Revised Edition.

Profusely illustrated with Original Cuts, Tables, &c.

Handsomely bound in leather.

6/- net, 6/3 post free.

*Of all Booksellers, or of*

THE SCIENTIFIC PRESS, LTD., 28 & 29 Southampton Street, Strand, London, W.C.

